# Connecticut Veterinary Medical Association

**Published:** 1891-07

**Authors:** R. S. Todd

**Affiliations:** Secretary


### SOCIETY PROCEEDINGS.

*The Connecticut Veterinary Medical Association.*—The annual meeting of the Connecticut Veterinary Medical Association was held at the Scovill House, Waterbury, on Tuesday June 2, 1891, at 8 o'clock in the evening.

The members present were E. R. Storrs, Chas. H. Smith, H. Whitney, N. Tibbals, Robt. Todd and Thomas Bland. Geo. B. Towne, D.V.S., and Mr. J. Schofield, a veterinary student, were also present.

The president, Dr. Bridges, not being present, Dr. Chas. H. Smith, Second Vice-President, took the chair.

Dr. E. R. Storrs proposed Dr. Geo. B. Towne for membership.

The following officers were elected for the ensuing year: Thomas Bland, President; Chas. H. Smith, First Vice-President; Harrison Whitney, Second Vice-President; Robt. Todd, Secretary; Nathan Tibbals, Treasurer. Board of Censors: E. C. Ross, Geo. Bridges, E. R. Storrs, Harrison Whitney and Nathan Tibbals.

The retiring President, Dr. Geo. Bridges, not being present on account of poor health, his address of greeting was read by the Secretary, as follows:

Gentlemen:

It gives me great pleasure to greet you at this, the close of another year. I congratulate you on the success of our Association; little by little, step by step we have increased our membership, we have become influential outside of the profession as well as in it, commanding the respect of the public and official bodies of the State until the Connecticut Veterinary Medical Association has grown from its infancy, and passed through the struggles of its first years, through the combined efforts of a determined few who saw its needs and usefulness in the future, and who—let me add—have stood fast to the helm ever since, and now we have a regularly chartered and incorporated association composed of a body of men who are determined to still farther advance the profession in our State, and at the same time are ever ready to protect stock-owners from losses through contagious and infectious diseases, and only ask the confidence of the public that they may also be the means of protecting the human family from the transmission of such diseases from animals to man.

There have been many very interesting papers read and much benefit derived from their discussion. Interesting and instructive cases have been freely quoted by all. Much has been accomplished, and last, but by no means least, the tie of fraternal fellowship has been still further strengthened and it is the earnest wish of your humble servant that nothing will ever disturb it.

We may have our little differences outside, but no matter, let us go on in the future as we have during the past.

Our financial standing is good and we are everyway in a flourishing condition. We have a Committee on Contagious and Infectious diseases who have accomplished good work during the past winter.

No doubt you have all read their report as published by the State Board of Health. It is a correct version of the condition existing in our State on such diseases and a step in the right direction. The committee are to be congratulated on this good work, and I know they have the thanks of every member of this profession at home and abroad, not speaking of the thanks due them from the many loving mothers and fathers who are many times innocently using milk from diseased cows. If you have not done so I would suggest that the report be struck off in pamphlet form and well distributed throughout the State.

I hope this committee will be continued, and if the political muddle ever clears up let them draft a good strong Bill.

If on tuberculosis I am in favor of inspection of all herds of dairy cattle; compelling the farmers to report all cases in their herds accompanied by a certificate of his attending veterinarian. Any neglect on his part to carry out the law to be amenable to punishment.

I would have a fine imposed on any veterinarian who refused to grant such certificate or failed to report any contagious disease of whatever nature brought to his notice. The question of indemnity to owners of diseased stock has often been discussed in different States, and it seems to be the opinion of many that a glandered horse is not worth anything anyway. I do not think that should be so. Why should a glandered horse be a dead loss in a State where this loathsome disease has been allowed to run rife for many years? In my opinion if this State would pay something, no matter how small, that in one year or two at the outside, with proper laws and proper enforcement, the disease would be entirely stamped out.

Before concluding, I would like to call your attention to one thing we have neglected in the past year. We have not performed our duty towards our American Veterinary JOURNALS. These journals must have support to live and material for publication. I would suggest that we give regularly papers that are read and those of the cases quoted. I do not think it a good plan to crowd their pages with every detail of these meetings, but as much as in the Secretary's judgment would be interesting to the profession. I know that I find much food for thought in the reports of other associations and I think it no more than right that we, too, should contribute our share.

In conclusion I wish to thank you for the great honor you conferred upon me in the past two years. As I look back on the time now passed away I cannot but think of it with pleasure. At each meeting every member carried himself with decorum becoming a gentleman.

I take this opportunity to thank our faithful Secretary for his courtesy towards me on all questions and at all times, and if I have in any way offended him by alluding to the above, I wish to assure him that if I were his judge I would certainly acquit him.

I am Yours Fraternally,

GEO. BRIDGES, D. V. S.

The next meeting will be held in New Haven, on Tuesday, September 1, 1891, at which time Messrs. Bland and Todd will read papers.

R. S. TODD, *Secretary*.

